# Consolidated bioprocessing for butanol production of cellulolytic Clostridia: development and optimization

**DOI:** 10.1111/1751-7915.13478

**Published:** 2019-08-26

**Authors:** Zhiqiang Wen, Qi Li, Jinle Liu, Mingjie Jin, Sheng Yang

**Affiliations:** ^1^ School of Environmental and Biological Engineering Nanjing University of Science and Technology Nanjing 210094 China; ^2^ College of Life Sciences Sichuan Normal University Longquan, Chengdu 610101 China; ^3^ Key Laboratory of Synthetic Biology CAS Center for Excellence in Molecular Plant Sciences Shanghai Institute of Plant Physiology and Ecology Chinese Academy of Sciences Shanghai 200032 China; ^4^ Huzhou Center of Industrial Biotechnology Shanghai Institutes of Biological Sciences Chinese Academy of Sciences Shanghai 200032 China

## Abstract

Butanol is an important bulk chemical, as well as a promising renewable gasoline substitute, that is commonly produced by solventogenic Clostridia. The main cost of cellulosic butanol fermentation is caused by cellulases that are required to saccharify lignocellulose, since solventogenic Clostridia cannot efficiently secrete cellulases. However, cellulolytic Clostridia can natively degrade lignocellulose and produce ethanol, acetate, butyrate and even butanol. Therefore, cellulolytic Clostridia offer an alternative to develop consolidated bioprocessing (CBP), which combines cellulase production, lignocellulose hydrolysis and co‐fermentation of hexose/pentose into butanol in one step. This review focuses on CBP advances for butanol production of cellulolytic Clostridia and various synthetic biotechnologies that drive these advances. Moreover, the efforts to optimize the CBP‐enabling cellulolytic Clostridia chassis are also discussed. These include the development of genetic tools, pentose metabolic engineering and the improvement of butanol tolerance. Designer cellulolytic Clostridia or consortium provide a promising approach and resource to accelerate future CBP for butanol production.

## Graphical abstract

This review focuses on consolidated bioprocessing (CBP) advances for butanol production of cellulolytic Clostridia and various synthetic biotechnologies that drive these advances. Moreover, the efforts to optimize the CBP‐enabling cellulolytic Clostridia chassis are also discussed.

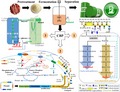

## Introduction

Butanol is a bulk chemical and a promising renewable vehicle fuel (Nguyen *et al*., 2018). Acetone–butanol–ethanol fermentation (with n‐butanol as the main product) by solventogenic Clostridia such as *Clostridium acetobutylicum* or *C. beijerinckii* has been developed more than a century ago (Moon *et al*., 2016). During the past 100 years, all aspects of butanol fermentation have progressed significantly, especially with regard to feedstocks. With increasing grain prices and a growing concern about food security by governments, the feedstocks for butanol fermentation are gradually expanding from corn to the less expensive, renewable lignocellulosic biomass and syngas (Gu *et al*., 2011, 2014; Jiang *et al*., 2015). Steel mill off‐gas is considered to be the most economic source and substrate; however, the performance of current gas‐fermenting strains for butanol production and their resistance to stress requires further modification (Durre, 2016; Huang *et al*., 2016, 2019). At present, butanol production by *Clostridium* from lignocellulosic biomass hydrolysate is very close to that from grain fermentation (Gu *et al*., 2011, 2014; Jiang *et al*., 2015); however, its industrialization bottleneck lies in the addition of exogenous cellulases. It has been estimated that the current cost of cellulases and pretreatment accounts for 35% of the overall cost (Jiang *et al*., 2015), thus weakening its market competitiveness.

Several steps are involved in microbial butanol production from lignocellulosic biomass including pretreatment, cellulase (cellulosome) secretion, lignocellulose enzymatic hydrolysis and co‐fermentation of hexose and pentose. Compared with other biorefinery processing such as separate hydrolysis and fermentation (SHF), simultaneous saccharification and fermentation (SSF), and simultaneous saccharification and co‐fermentation (SSCF), consolidated bioprocessing (CBP) combines all above steps in one reactor, thus decreasing the cost from dedicated cellulase production (Lynd *et al*., 2005; Olson *et al*., 2012; den Haan *et al*., 2015). CBP is considered a promising biorefinery process and has been adapted for cellulosic butanol production, which achieved notable progress (Xin *et al*., 2019).

The recent progress of synthetic biology technology in *Clostridium* provides an abundance of tools and approaches towards to realization of CBP (Joseph *et al*., 2018; McAllister and Sorg, 2019), which effectively drive butanol production by CBP. For example, genome editing and metabolic engineering have been used to modify and optimize CBP‐enabling strains or consortia (Wen *et al*., 2017, 2019). Omics analysis (Patakova *et al*., 2018; Liu *et al*., 2019), metabolic and dynamic modelling (Salimi *et al*., 2010; Liao *et al*., 2015) and other techniques have been used to analyse butanol tolerance or to optimize the community structure of producers.

This review intends to highlight the CBP progress based on cellulolytic clostridia driven by synthetic biology technique. Three different synthetic biological approaches to realize CBP are described and compared, with particular focus on the progresses of butanol metabolic engineering in native cellulolytic Clostridia, designer cellulosome secretion and optimization of clostridial consortia. Progress towards overcoming a number of bottlenecks for butanol production by CBP including the development of genetic tools, xylose utilization and butanol tolerance is also discussed.

## CBP development based on cellulolytic Clostridia

Consolidated bioprocessing requires microorganisms or microbial systems capable of degrading lignocellulose, while efficiently and simultaneously yielding chemicals. However, few native microorganisms or microbial systems can directly degrade lignocellulose and produce butanol (Wen *et al*., 2017, 2019).

**Figure 1:**
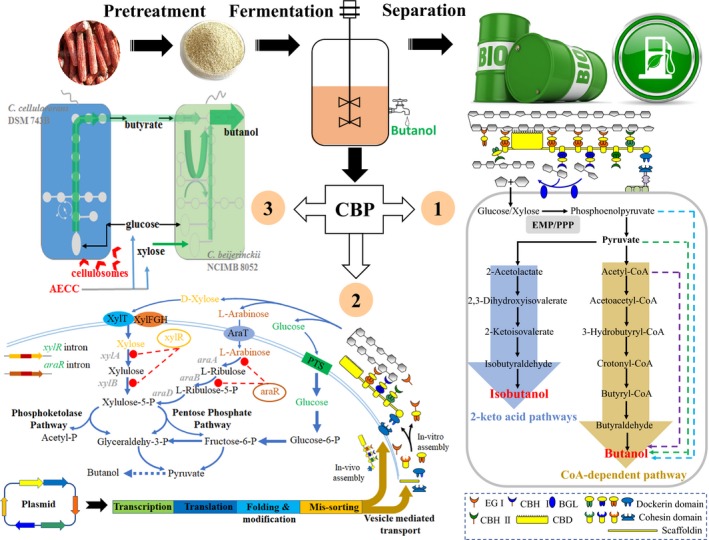
CBP approaches based on cellulolytic clostridia or consortia for butanol production.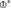, Butanol/isobutanol pathway engineering of cellulolytic clostridia. Purple dotted line of represents ACP dependent pathway (Pasztor, *et al*., 2015), while green and blue dotted lines represent 2‐keto acid pathway extended from pyruvate and phosphoenolpyruvate (Chen and Liao, 2016) respectively;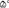, cellulosome or cellulases overexpression of butanol‐producing clostridia. EGI, endoglucanase; CBHI, cellobiohydrolase I; BGL, β‐glucosidase; CBHII, cellobiohydrolase II; CBD, cellulose binding domain; 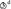, engineering of consortia composing of cellulolytic and butanol‐producing clostridia, taken a twin‐clostridia consortium as example(Wen *et al*., 2017). In the consortium, *C. cellulovorans* secretes cellulosome to degradate AECC (alkali extracted corn cobs) to provide glucose and xylose for *C. beijerinckii* to grow and produce butanol; besides, butyrate produced by *C. cellulovorans* can be re‐assiminated by *C. beijerinckii* to produce butanol.

Currently, three strategies are used to achieve CBP (Higashide *et al*., 2011; Zuroff and Curtis, 2012; den Haan *et al*., 2015). These include butanol pathway engineering of cellulolytic Clostridia, engineering solventogenic Clostridia to secrete/cell display cellulases or cellulosomes and mixed‐culture of cellulolytic and butanol‐producing Clostridia (Fig. 1). Each strategy has its unique advantages and together, and they promote the study of butanol production by CBP.

### Engineering the butanol pathway in cellulolytic Clostridia

Several Clostridia natively secrete cellulases and directly grow on lignocellulose with alcohols and organic acids as main products. Examples are *C. thermocellum* (ethanol, acetate and lactate) (Ng *et al*., 1977), *C. cellulolyticum* (ethanol, acetate and lactate) (Petitdemange *et al*., 1984) and *C. cellulovorans* (butyrate, ethanol and lactate) (Sleat *et al*., 1984). Whole genome sequencing and annotation have deepened the current understanding of lignocellulose‐degrading enzymes and butanol metabolic networks of the above cellulolytic Clostridia. Clostridial genetic manipulation techniques provide an opportunity to engineer cellulolytic Clostridia for n‐butanol/isobutanol production by CBP (Wen *et al*., 2017; Joseph *et al*., 2018).

*Clostridium cellulolyticum* has been modified to extend the CoA‐dependent metabolic pathway for n‐butanol production; however, the titre is < 0.12 g l^−1^ (Gaida *et al*., 2016). Higashide *et al*. (2011) introduced a hybrid valine biosynthesis pathway in *C. cellulolyticum* and then diverted its 2‐keto acid intermediates towards isobutanol synthesis. This was the first successful report on isobutanol production via metabolic engineering of cellulolytic clostridia; however, only 0.66 g l^−1^ of isobutanol was produced within 192 h. Recently, *C. thermocellum* was engineered by the same research group to extend the 2‐keto acid metabolic pathway for isobutanol synthesis (Lin *et al*., 2015), and finally, 5.4 g l^−1^ isobutanol was produced from cellulose within 75 h. This notably improved titre and productivity, implying promising potential of *C. thermocellum* for CBP.

Similar to *C. thermocellum*,* C. cellulovorans* is a further promising cellulolytic clostridial chassis for butanol production, since it can grow on lignocellulose and accumulate a high amount of butyrate, the precursor of butanol (Sleat *et al*., 1984). Yang *et al*. (2015) overexpressed the aldehyde/alcohol dehydrogenase gene (*adhE2*) in *C. cellulovorans,* which caused the recombinant strain to produce 1.42 g l^−1^ n‐butanol within 252 h. This exemplified the first metabolic engineering in *C. cellulovorans*, but the complicated genetic modification has remained unexplored due to a lack of genetic tools. Wen *et al*. (2017) verified that genetic tools such as TargeTron and the CRISPR/Cas system were applicable in *C. cellulovorans*. Based on efficient genetic techniques, a CoA‐dependent acetone–butanol–ethanol (ABE) pathway was introduced into *C. cellulovorans* to direct carbon flux from butyrate to n‐butanol. To further improve n‐butanol production, strains with high butanol tolerance were adapted as hosts for metabolic engineering. The generated strains produced 3.47 g l^−1^ of n‐butanol, which is 139‐fold of that produced by wild‐type *C. cellulovorans* (Wen *et al*., 2019). Recently, n‐butanol production by *C. cellulovorans* achieved 4.0 g l^−1^ by Bao *et al*. (2019).

These studies demonstrated that cellulolytic Clostridia are ideal strain platforms for biorefinery by CBP. However, it still remains challenging to simultaneously balance the capacity of efficient cellulose hydrolysis and butanol synthesis in a single strain (Song *et al*., 2014; Wen *et al*., 2017). The reason is that the modification of complicated multiple genes is sometimes delayed by the lack of genetic tools (Wen *et al*., 2019). Moreover, low titre, productivity and yield may also be related to insufficient carbon flux supply (due to inefficient lignocellulolytic hydrolysis) and competing metabolic pathways (Higashide *et al*., 2011; Yang *et al*., 2015). Further enhancement of cellulosome and/or cellulases expression and butanol metabolic flux is expected to significantly increase n‐butanol/isobutanol production.

### Overexpression of cellulosome or cellulases in butanol‐producing Clostridia

Although several butanol‐producing Clostridia can secrete xylanase and degrade xylan, they lack major lignocellulose‐degrading enzymes (such as endoglucanase, exoglycanase and cellobiohydrolase) (Thomas *et al*., 2014; Jiang *et al*., 2018a,b). Basically, Clostridia cannot directly grow on lignocellulose. Fortunately, butanol‐producing Clostridia are taxonomically closely related to many cellulolytic Clostridia and have similar codon preference, which laid the foundation for the heterologous expression of cellulase genes in butanol‐producing Clostridia.

The expression of soluble cellulases with small molecular weight (such as Cel5A, Cel8C and Cel9M) in *C. acetobutylicum* has been successfully realized (Lopez‐Contreras *et al*., 2003, 2004; Mingardon *et al*., 2011). However, single type cellulase and very low expression levels (0.5‐5 mg l^−1^, only detectable cellulases activity) cannot support the host to directly utilize lignocellulose for growth and butanol production.

Compared with free and single component cellulase, cellulosome (a multi‐enzyme complex) typically achieved much better results. The reason is that cellulosome can adhere to the lignocellulose surface, which contributed to multi‐enzyme synergy and locally maintains a relatively high concentration of cellulases (Bayer *et al*., 2007). Interestingly, although complete gene clusters of cellulosome have been identified in a number of Clostridia (e.g. *C. acetobutylicum*), very small quantities of functional cellulosome were produced (Sabathe *et al*., 2002).

Currently, studies mainly focus on the development of hybrid cellulosomes and the optimization of heterologous expression. Heterologous production, assembly and secretion of a mini‐cellulosome by *C. acetobutylicum* ATCC 824 was first reported by Mingardon *et al*. in 2005. Subsequently, a number of key issues have been addressed towards the secretion of heterologous cellulosome and cellulases, including the investigation and optimization of leader peptide, promoter strength and molecular chaperones (Mingardon *et al*., 2011; Fierobe *et al*., 2012; Hyeon *et al*., 2013). Recently, allele‐coupled exchange (ACE) technology was adapted to stably integrate a hybrid cellulosome operon composed of cellulosomal enzymes and miniscaffolding genes into chromosomes (Kovács *et al*., 2013; Willson *et al*., 2016). Furthermore, the secreted mini‐cellulosome was successfully anchored to the *C. acetobutylicum* cell wall via the native sortase system (Willson *et al*., 2016). The thus generated recombinant strains displayed an improved growth phenotype on lignocellulosic biomass (Willson *et al*., 2016). This represents a milestone towards enabling solventogenic Clostridia to be cellulolytic.

However, the number of recombinant solventogenic Clostridia that harboured the overexpression of cellulases or hybrid (chimeric) cellulosomes and that can grow on lignocellulose as the sole carbon source is limited (Wen *et al*., 2017). Many technical challenges still need to be overcome towards cellulases expression. For example, regulation of selective RNA processing and stabilization on the cellulosome operon indicates the importance of post‐transcriptional modification studies (Xu *et al*., 2015a,b,c). In addition, confirmation of the correct translation, folding and efficient transmembrane transport of cellulases and designer cellulosomes with high molecular weight and complex structure still remain challenging. This aggravates the simultaneous efficient lignocellulose degradation and butanol fermentation (Mingardon *et al*., 2011).

### Engineering consortia composed of cellulolytic and solventogenic Clostridia

Mixed‐culture of cellulolytic and butanol‐producing Clostridia towards the construction of a cross‐species cellulosic butanol pathway can share the metabolic requirements and adjust the abundance of each strain to adapt to the external environment (Song *et al*., 2014). Moreover, it is advantageous to explore and improve beneficial microbial interactions (Wen *et al*., 2017) and thus promote butanol production. Consortia engineering has become an emerging strategy for butanol production by CBP (Zuroff and Curtis, 2012; Xin *et al*., 2019).

Many commensal consortia have been isolated from nature for butanol production from lignocellulose (Jiang *et al*., 2018a,b); however, it remains difficult to regulate these systems due to their complex community structure and the limitation of available genetic tools. Compared with natural consortia, artificial consortia are relatively simple to construct and regulate.

The sequential co‐culture of thermophilic *C. thermocellum* and mesophilic *C. acetobutylicum*,* C. saccharoperbutylacetonicum* N1‐4 or *C. beijerinckii* was designed to directly produce n‐butanol from cellulose (Yu *et al*., 1985; Nakayama *et al*., 2011; Wen *et al*., 2014a,b). However, the un‐matched incubation temperature resulted in a non‐isothermal and long fermentation process and, thus, low productivity. Therefore, it is critical to select appropriate strains for the formation of a consortium.

Mesophilic cellulolytic Clostridia, such as *C. cellulolytium* and *C. cellulovorans*, have been adapted to replace *C. thermocellum* (Petitdemange *et al*., 1983; Wen *et al*., 2014a,b). In *C. cellulovorans* and *C. beijerinckii* co‐culture, *C. cellulovorans* degrades lignocellulose and provides fermentable sugars to support *C. beijerinckii*'s growth and solvent fermentation; in turn, *C. beijerinckii* re‐assimilates and detoxicates butyrate for *C. cellulovorans*. A novel feeding‐detoxification relationship between *C. cellulovorans* and *C. beijerinckii* was developed in the multicellular system. Their symbiosis degraded 68.6 g l^−1^ of alkali extracted deshelled corn cobs (AECC) and produced 8.30 g l^−1^ n‐butanol within 80 h, which demonstrated the promising potential of synthetic consortia in the biorefinery (Wen *et al*., 2014a,b).

However, the lack of genetic techniques that enable *C. cellulovorans* to be used forms a main obstacle of the further improvement of the twin‐Clostridia consortia (Wen *et al*., 2014a,b). Recently, Wen *et al*. (2017) developed an electroporation protocol and demonstrated that several genetic tools, such as TargeTron and the CRISPRi system, are functional in *C. cellulovorans*. Based on available genetic techniques, the overall cross‐species n‐butanol synthesis pathway in the consortia can be split into four modules. The toxic intermediates (butyrate) metabolism module and detoxification module are installed into *C. cellulovorans* and *C. beijerinckii*, respectively, which strengthens their feeding‐detoxification relationship. Multivariate modular metabolic engineering was adapted to promote butyrate delivery towards the enhancement of n‐butanol production. The engineered twin‐Clostridia consortia decomposed 83.2 g l^−1^ AECC and produced 11.5 g l^−1^ n‐butanol, the titre of which approximated ABE output from starchy feedstocks (Wen *et al*., 2017).

The performance of synthetic consortia significantly depends on the benefit of the interaction between strain species (Song *et al*., 2014), which can be further improved via genetic engineering of strains and optimization of culture conditions. However, the empirical strategy needs to be combined with model‐driven analysis (Salimi *et al*., 2010; Yoo *et al*., 2015; Zomorrodi and Segre, 2016), to rationally design and regulate the synthetic consortia towards increased efficiency and robustness (Zomorrodi and Segre, 2016).

Butanol production with varied lignocellulosic feedstocks by CBP is depicted in Table 1. In general, artificial consortia provide a more convenient and feasible approach for butanol production via synergistic utilization of the metabolic pathways of cellulolytic and solventogenic Clostridia. Furthermore, although differences in feedstock exist, the engineered synthetic consortia offered much higher butanol titres and productivity overall, compared with co‐culture with wild‐type strains and pure culture of recombinant bacteria, including engineered cellulolytic and solventogenic Clostridia. However, it should be pointed out that pure culture is comparatively simple for genetic modification and optimization of culture conditions. These three approaches have the potential to realize large‐scale butanol production by CBP. Challenges and bottlenecks of different approaches will likely be resolved by advances in synthetic biology techniques.

**Table 1:** Comparison of butanol production by different CBP approaches and substrates

Strain/consortium	CBP approaches	Genotype	Substrate	Titre (g l^−1^)	Productivity (g l^−1^ h^−1^)	Mode	References
*C. cellulolyticum* ATCC 35319	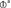	+ *kivd yqhD alsS ilvCD*	Crystalline cellulose	0.66^b^	0.0031	Batch	Higashide *et al*. (2011)
*C. cellulolyticum* ATCC 35319	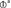	+*ato hbd crt bcd‐adhE2*	Crystalline cellulose	0.12	.00025	Batch	Gaida *et al*. (2016)
*C. thermocellum* DSM 1313	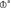	*Δhpt+ilvBNCD kivD*	Crystalline cellulose	5.4^b^	0.072	Batch	Lin *et al*. (2015)
*C. cellulovoran* DSM 743B	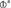	+*adhE2*	Crystalline cellulose	1.42	0.0056	Batch	Yang *et al*. (2015)
*C. cellulovoran* DSM 743B	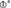	+*adhE2*	Pretreated corn cob	3.36	0.028	Batch	Ou *et al*. (2017)
*C. cellulovoran* DSM 743B	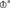	+*adhE2*	Crystalline cellulose	4.0	0.0128	Batch	Bao *et al*. (2019)
*C. cellulovoran* DSM 743B	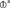	Clocel^e^: +*adhE1 ctfAB‐adc*	Alkali extracted corn cobs	3.47	0.0413	Batch	Wen *et al*. (2019)
*C. acetobutylicum* ATCC 824	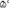	*+Cel5A, +Cel8C, +Cel9M*	Crystalline cellulose	ND	ND	Batch	Mingardon *et al*. (2011)
*C. beijerinckii* NCIMB 8052	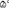	+*celA*, +*celD*	Microcrystalline cellulose	ND	ND	Batch	Lopez‐Contreras *et al*. (2001)
*C. acetobutylicum* ATCC 824	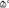	+*Cel8A Cel9B CipA variants*	Cellohexaose	ND	ND	Batch	Kovács *et al*. (2013)
*C. acetobutylicum* ATCC 824	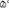	*+Cel9G Cel48F Xyn10A CipC*	Untreated wheat straw	ND	ND	Batch	Willson *et al*. (2016)
*C. thermocellum* and *C. saccharoperbutylacetonicum* strain N1‐4	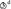	Wild type	Crystalline cellulose	7.9	0.0299	Batch	Shunichi *et al*. (2011)
C. celevecrescens N3‐2 and C. *acetobutylicum* ATCC 824	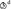	Wild type	Filter paper	2.69	0.014	Batch	Wang *et al*. (2015)
*C. thermocellum* ATCC 27405 and *C. beijerinckii* NCIMB 8052	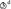	Wild type	Alkali extracted corn cobs	10.9	0.0556	Fed‐batch	Wen *et al*. (2014a,b)
*C. cellulovorans* DSM 743B and *C. beijerinckii* NCIMB 8052	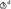	Wild type	Alkali extracted corn cobs	8.3	0.106	Fed‐batch	Wen *et al*. (2014a,b)
*C. cellulovorans* DSM 743B and *C. beijerinckii* NCIMB 8052	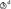	Clocel: +*buk*△*ldh*△*ack*△*hydA*; Cbei: +*xylT ctfAB*△*xylR*	Alkali extracted corn cobs	11.8	0.0983	Fed‐batch	Wen *et al*. (2017)

Clocel, *C. cellulovorans*; Cbei, *C. beijerinckii*; ND, not detected*;* +, overexpression; Δ, deficient or inactivation.

**a**. Metabolic engineering of cellulolytic Clostridia.

**b**. Titre of isobutanol.

**c**. Cellulase expression of butanol‐producing Clostridia.

**d**. Clostridia consortia engineering.

**e**. Evolved strain.

## CBP optimization by cellulolytic Clostridia chassis engineering

Although CBP development has achieved great progress, a number of putative challenges remain that should be addressed. Current CBP‐enabling strains or consortia typically exhibit low butanol production, productivity and yield (Wen *et al*., 2014a,b, 2019). Their further optimization is often delayed by a lack of efficient genetic tools for complex genetic modification (Wen *et al*., 2017). In addition, the low butanol tolerance of *Clostridium* determines the upper limit of butanol production (Patakova *et al*., 2018), while inefficient xylose utilization also negatively influences hemicellulose degradation and conversion (Gu *et al*., 2011, 2014). Driven by advancements of clostridial synthetic biotechnology, progress has been made in the improvement of cellulolytic clostridia chassis to optimize CBP.

### Development of genetic tools

In the post‐genome era, synthetic biology techniques provide resources and approaches for CBP construction and optimization. However, inefficient DNA repair and low plasmid transformation efficiency lead to lack of synthetic biology tools for *Clostridium* (Li *et al*., 2016a,b), which severely delays CBP development. To accelerate the bottleneck breakthrough, roadmaps or technical guides for the genetic advancement in *Clostridium* have been extensively suggested and reviewed (Pyne *et al*., 2014; Minton *et al*., 2016; Joseph *et al*., 2018).

**Figure 2:**
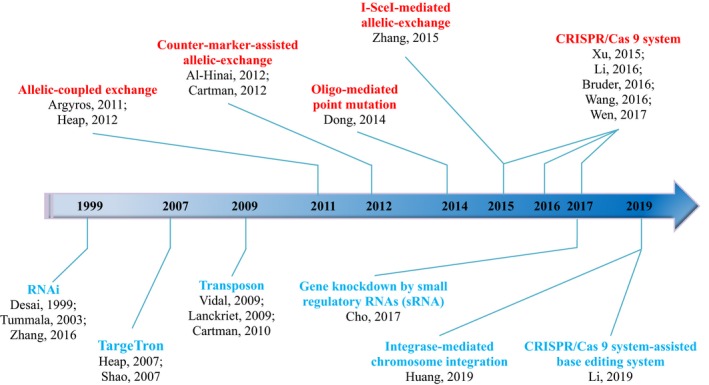
Genetic manipulation tools applicable for clostridia. Red bond, genetic tools dependent on homologous recombination; blue bond, genetic tools independent on homologous recombination.

Milestone studies of the development of genetic manipulation tools are shown in Fig. 2. Before the successful application of the CRISPR/Cas system to *Clostridium*, the TargeTron technology was preferred, because it is dependent on the site‐specific insertion of mobile group II introns rather than on homologous recombination (Cui and Davis, 2007; Rodriguez *et al*., 2009). This system is very suitable for genetic modification of bacteria and was considered intractable to conventional genetic tools. Mesophilic‐TargeTron and thermo‐TargeTron technology applicable in *Clostridia* have been developed based on Group IIA intron Ll. LtrB, as well as group IIB intron TeI3c and TeI4c respectively (Heap *et al*., 2007; Shao *et al*., 2007; Mohr *et al*., 2013). These have been applied in many *Clostridia* for metabolic engineering and function gene identification due to the manipulation convenience and high reliability they offer (Enyeart *et al*., 2014; Pyne *et al*., 2014; Liu *et al*., 2015). However, a number of native drawbacks exist in the Targetron technology such as incomplete inactivation, off‐target and polar effect with low frequency (Pyne *et al*., 2014; Liu *et al*., 2015a; Joseph *et al*., 2018).

Allelic‐exchange via single/double‐crossover is a classic genetic tool for precision manipulation based on homologous recombination (Argyros *et al*., 2011; Heap *et al*., 2012). This tool can realize in‐frame deletion or insertion of target genes. The only obstacle is the very low frequency to obtain positive single‐crossover or double‐crossover mutants, due to inefficient homologous recombination and low plasmid transformation efficiency of *Clostridium* (Argyros *et al*., 2011; Cartman *et al*., 2012). Although counter‐selection markers such as *mazF*,* tdc* (Al‐Hinai *et al*., 2012; Cartman *et al*., 2012) and endonuclease I‐SceI (Zhang *et al*., 2015) were applied to facilitate the screening of mutant yield during the second exchange, this still suffers from the long operation cycle and low first single‐crossover efficiency.

Since 2015, the CRISPR‐Cas system has been adapted to increase the frequency of mutants with single‐ or double‐crossover (Wang *et al*., 2015). However, the low homologous recombination efficiency indicates that DNA double‐strand breaks (DSB) cannot be repaired (Koh *et al*., 2014; Sun *et al*., 2017); moreover, the low plasmid transformation efficiency further negatively affects the screening of positive transformants. To overcome this particular problem, Xu *et al*. (2015a,b,c) and Li *et al*. (2016a,b) introduced Cas9 nickase to *C. cellulolyticum* and solventogenic Clostridia, respectively, which resulted in a single nick, which triggered homologous recombination and enhanced genome editing efficiency.

Furthermore, Li *et al*. (2019) developed a base editing system based on cytidine deaminase (rat Apobec1), uracil DNA glycosylase inhibitor (UGI) and CRISPR‐Cas9^D10A^ system. Apobec1 and UGI can efficiently convert specific C·G nucleotide base pairs within the CRISPR‐Cas9 targeting window sequence to T·A, which can create either missense mutations or null mutations in a gene. It is as precise as Cas9‐mediated genome editing, but it does not require DNA cleavage. Therefore, it requires no DNA repair templates. It is very simple to design and theoretically programmable to target all genes in *Clostridium*.

Except for genome editing, the CRISPR‐Cas system can also be used to regulate gene expression, which is similar to classical antisense RNA technology (Desai and Papoutsakis, 1999; Tummala *et al*., 2003), as well as the recent emergence of sRNA‐based gene downregulation techniques (Little *et al*., 2018). The CRISPR‐Cas system can also be combined with traditional transposon technology, which introduces random insertion inactivation into the genome by transposase and constructs a mutant library for screening for specific phenotypes (Lanckriet *et al*., 2009; Vidal *et al*., 2009; Cartman and Minton, 2010). More recently, Strecker and Ladha (2019) developed a RNA‐guided DNA insertion method that uses CRISPR‐associated transposases in *Escherichia coli*. These can be introduced into *Clostridium* and facilitate the development of programmable genetic manipulation tools based on non‐homologous recombination.

In general, the CRISPR‐Cas system is a disruptive technology that almost completely rewrites the patterns of genetic manipulation technology development in *Clostridium*. However, the homologous recombination capacity of *Clostridium* itself still requires further improvement by introducing genetic parts such as RecT (Dong *et al*., 2014).

### Pentose metabolic engineering

Most Clostridia cannot efficiently utilize pentose (mainly xylose) due to carbon catabolite repression (CCR) (Bruder *et al*., 2015; Mitchell, 2016), which severely reduces the butanol yield from lignocellulosic biomass, because xylose is the second‐most abundant sugar of the lignocellulosic hydrolysates (Jin *et al*., 2015). The first step to improve the efficiency of xylose uptake is to understand the CCR system, xylose metabolic pathways and transporters in Clostridium. The next step is their modification, as shown in Fig. 1.

Carbon catabolite repression is mediated by the catabolite control protein A (CcpA), which can form a complex with a serine‐phosphorylated histone‐containing protein (HPr) (Warner and Lolkema, 2003). The resulting complex binds at catabolite responsive element (CRE) sites within the promoter region or the coding sequence of transcriptional units to inhibit transcription (Lorca *et al*., 2005). Inactivation of *ccpA* or *glcG* (encoding EII of the phosphotransferase system (PTS)) can overcome xylose metabolism inhibition (Ren *et al*., 2010; Xiao *et al*., 2011). Refined modification such as generation of a *ccpA* mutant, deficient in co‐effector HPr‐Ser^46^‐P binding (Wu *et al*., 2015), repression of *hprK* gene (encoding HPr phosphorylase/kinase, HPrK) by CRISPR/dCas9 (Bruder *et al*., 2016) or elimination of CcpA binding sites in xylose and pentose phosphate pathway operon (Bruder *et al*., 2015) have also been demonstrated. These worked well in the simultaneous utilization of xylose and glucose.

The xylose metabolism is also regulated by other mechanisms. Hu *et al*. (2011) and Xiao *et al*. (2011) identified putative transcriptional regulators such as *CEA_G2622* and *Cbei_2385* (*xylR*), which are involved in the xylose metabolism in *C. acetobutylicum* EA 2018 and *C. beijerinckii* NCIMB 8052 respectively. The inactivation of *xylR* or *araR* can upregulate the transcription of the xylose isomerase gene (*xylA‐II*,* CAC2610* and xylose isomerase) and the xylose kinase gene (*xylB* and *CAC2612*), thus promoting the utilization of the xylose metabolism.

Gu *et al*. (2010) identified several key genes involved in xylose metabolism in *C. acetobutylicum* using TargeTron combined with other genetic and biochemical methods. The xylose metabolic pathway was reconstructed in *C. acetobutylicum* according to previous studies and comparative genomic predictions. Subsequently, Liu *et al*. (2012) reported how *C. acetobutylicum* metabolizes xylose using both the pentose phosphate pathway and the phosphoketolase pathway via isotope tracing techniques. Based on this, xylose utilization efficiency could be significantly improved by overexpressing genes involved in the xylose metabolic pathway (Gu *et al*., 2009; Xiao *et al*., 2011, 2012).

Furthermore, transporter engineering has also achieved great progress. Xiao *et al*. (2012) identified a D‐xylose proton‐symporter (encoded by gene *cbei‐0109*) and overexpressed it to enhance the xylose uptake in *C. beijerinckii*. Recently, Sun *et al*. (2015) demonstrated that the hexa‐protein module XylFII‐LytS/YesN‐XylFGH is associated with xylose utilization in *C. beijerinckii*. Li *et al*. (2017) further confirmed that it is a new ‘three‐component’ xylose response and regulatory system whose molecular mechanisms were deeply analysed.

The above‐mentioned xylose metabolism‐related functional genes basically mapped the framework of pentose transport, metabolism and regulation in solventogenic Clostridia (Fig. 1). Studies of xylose metabolic engineering in solventogenic Clostridia provide a reference for cellulolytic clostridia used in CBP (Xiong *et al*., 2018).

### Improvement of butanol tolerance

Butanol toxicity is considered a major barrier for butanol accumulation in fermentation media to high titre since this might cause microbial growth inhibition and cell death (Papoutsakis, 2008; Patakova *et al*., 2018). To date, n‐butanol output did not exceed 21 g l^−1^, which was achieved by *C. beijerinckii* BA101in 1999 (Chen and Blaschek, 1999). A low butanol titre increases the cost of product separation, which impedes the economics of the CBP process.

Traditional random mutations and optimization of culture methods have often been adapted to obtain high butanol‐producing and tolerant strains, such as BA101, GS4‐3, SA‐1 and BKM19 (Chen and Blaschek, 1999; Jang *et al*., 2013; Sandoval‐Espinola *et al*., 2013; Li *et al*., 2016a,b). These provide an opportunity to observe, describe or hypothesize the butanol tolerance mechanism.

Recently, many synthetic biological techniques, such as comparative genomics, transcriptomics, metabolomics and proteomics, have attempted to analyse the mechanisms by which Clostridia respond to butanol stress (and to identify putative gene targets). The complex responses are reflected in the aspects of overall cell, cell envelope and cytoplasm (Liu *et al*., 2017; Patakova *et al*., 2018), including cell wall and cell membrane modifications (*Cfa*, a cyclopropane fatty acid synthase gene (Zhao *et al*., 2003) and *Lyt‐1*, a cell wall lytic enzymes gene (Van Der Westhuizen *et al*., 1982)), stress protein production (*GroESL* and *DnaK* (Jones *et al*., 2016; Liao *et al*., 2017)), butanol excretion by efflux pumps (formate transporter *FocA* (Reyes *et al*., 2011) and multidrug efflux pump *AcrB* (Fisher *et al*., 2014)), and other mechanisms involved in n‐butanol tolerance (AdhE mutation (Tian *et al*., 2019) and Spo0A overexpression (Alsaker *et al*., 2004)).

Based on the above known or not yet fully understood mechanisms (and gene targets), synthetic biology strategies have been introduced to enhance butanol tolerance and thus further understand the underlying mechanisms. Xu *et al*. (2015a,b,c) obtained the *C. acetobutylicum* mutant JB200 with high butanol production of 21 g l^−1^ through evolution in a fibrous bed bioreactor (FBB). Comparative genomic analysis and reverse metabolic engineering confirmed that the *cac_3319* gene encodes histidine kinase (HK), which is responsible for the enhanced n‐butanol tolerance. In a further adaptation study in a chemostat, Tian *et al*. (2019) showed that a D494G mutation in the *adhE* gene or *adhE* deletion can enhance the butanol tolerance of *C. thermocellum* from 5 to 15 g l^−1^.

More recently, Wen *et al*. (2019) developed a novel adaptive laboratory evolution (ALE) approach based on *spo0A*‐deficient strains to enhance the positive ratio of evolved mutants. The generated strain can tolerate 12 g l^−1^ n‐butanol. Moreover, Wen *et al*. adapted the evolved strain as host for further metabolic engineering, which achieved much higher butanol production than using the wild‐type strain as chassis for metabolic engineering. This indicates that the butanol tolerance enhancement of Clostridium can promote butanol production (Nicolaou *et al*., 2010). However, it must be pointed out that butanol tolerance and butanol production are not always tightly associated (Liu *et al*., 2013). Butanol tolerance only determines the upper limit of butanol production and is not the decisive factor for high butanol production.

## Conclusions and outlook

Consolidated bioprocessing is the logic end of the evolution of biorefinery routes towards the production of cellulosic butanol. Here, cellulolytic Clostridia are more important than saccharolytic and gas‐fermenting *Clostridium* species. CBP development based on cellulolytic Clostridia has achieved great progress in the aspects of butanol pathway engineering of native cellulolytic clostridia. This enables solventogenic Clostridia to be cellulolytic, as well as the engineering of consortia composed of cellulolytic and butanol‐producing Clostridia. However, improvement and optimization of CBP‐enabling Clostridia (consortia) have been delayed due to a lack of efficient genetic tools. Moreover, low butanol tolerance and inefficient xylose utilization in Clostridia negatively affect butanol titre and yield respectively. In addition to these bottlenecks, many other problems need to be addressed, such as hydrolysate inhibitors resistance, balance of species population competition and cooperation.

Recently, advances have been achieved in the study of synthetic biotechnology accelerated CBP based on cellulolytic Clostridia. For instance, programmable genome editing tool‐assisted metabolic engineering provided a variety of reliable approaches and resources for the construction and optimization of designer cellulosomes, microbes or consortia. Moreover, omics data (genome, transcriptome, proteome and metabolome) and various models (genome‐scale metabolic network models (GSSM), kinetic models and population interaction models) will promote the design and re‐construction of CBP‐enabling Clostridia. CBP driven by clostridial synthetic biotechnology is a promising technique to achieve large‐scale production of cellulosic butanol in the near future.

## Conflict of interest

None declared.
